# Sulphate of Cinchona Equal to Sulphate of Quinia, in the Treatment of Intermittents

**Published:** 1856-11

**Authors:** L. Faulkner

**Affiliations:** Halifax County, Va.


*Sulphate of Cinchona equal to Sulphate of Quinia, in the Treatment of Intermittents.* By L. FAULKNER, M.D. Halifax County, Va.

Old-fogyism is surely often met with in the medical profession, especially, I am inclined to suspect, in Virginia. What else prevents the substitution of Sulphate of Cinchona for sulphate of quinia, but the old habit of going to mill with a bushel of corn in one end of the bag and a stone in the other? That new remedies should be used with caution, all will admit; but when a substitute is recommended to us by so many considerations, it is the imperative duty of practitioners to try it, and introduce it into practice, if proved worthy; and its price being only one-half that of a costly and frequently-used medicine, will justify its substitution in many cases, even if found of inferior though similar properties.

The use, in practice, of an active principle of Peruvian bark, heretofore thrown away, and which is obtained in large quantities from a class of barks entirely rejected for want of a good per centage of quinia, not only affords a cheaper substitute in itself, but must evidently diminish the price of quinia, by curtailing the demand. It is far from being a question of dollars and cents merely to the physician or even to the people. It is a question of life or death to many. In such an epidemic as prevailed in this county from 1843 to 1848—at its acme in 1846—when it overspread the whole community, leaving out scarcely a family, or a member of a family—the sulphate of cinchona, whilst saving, doubtless, *thousands* of dollars to the county, would, I have every reason to believe, have prevented many shattered constitutions, and perhaps some deaths, which, however, were rare. Every physician engaged in practice knows, that not only the poor suffer for the want of quinine, or are thrown upon his charity, but many, who are independent livers, in times of great sickness from intermittents, use quinine with stint and of inferior quality.

Sulphate of cinchona cannot now be regarded purely as an experimental medicine. Occasional successful experiments have been made with it ever since 1821; and Pereira says, whilst it fell into disuse principally upon the evidence of Chomel, "the subsequent observations of Dufour, Petroz, Potier, Bally, Nieuwenhuiss, Mariani, Bleynie, and others have proved that the di-sulphates of these alkalies (quinia and cinchona) may be substituted for each other. Nay, Bally gives the preference to the di-sulphate of cinchona, on the ground that it is less irritating than the di-sulphate of quinia." Prof. Wood says, "there is little doubt, however, that cinchona possesses febrifuge properties little, if at all, inferior to those of quinia." Dr. Pepper, in the *American Journal*, January, 1853, reports 17 cases of intermittents satisfactorily treated with it in the Pennsylvania Hospital. Dr. R. P. Thomas, in the College of Physicians of Philadelphia, as may be seen in same journal, January, 1856, after referring to the experiments of Drs. Pepper and Gerhard, and quoting from *Ranking's Abstract*, for June, 1855, that M. Hudelet, physician to the hospital at Bourg, since March, 1853, out of 507 cases of intermittents of all kinds, had cured all except 9 with this remedy, states that, at his instance, in the Western Clinical Infirmary, all except 1 in 10 cases of remittent, and 109 of intermittent had been cured with it; and in the Philadelphia Dispensary, 102 walking cases of intermittent have been treated with it, and the results carefully observed by Dr. George Martin, who has prepared tables exhibiting the results, comparing very favorably at least with those of quinine. And, in the *Cincinnati Medical Observer*, January, 1856, as quoted in the *American Journal*, April, 1856, Dr. J. C. Wells reports 53 out of 57 cases of intermittent cured with it at the City Dispensary.

To add my feeble testimony to this, which has been hurriedly referred to in a few minutes in a scanty library, I will simply give the result, without the notes in detail, of the treatment of seven cases of intermittent with sulphate of cinchona, omitting an eighth, which has not been heard from, and a ninth, which took one dose of two grains just at the period of accession of chill, and afterwards was put upon quinine. I used it in the same quantities that all of us do of quinia, generally giving our minimum doses, and was always particular in weighing it instead of guessing at the dose, lest I might be deceived by its smaller bulk, which, by the way, is one of its advantages. The specimen used was sent me, at my request, by Dr. James Cooke & Co. Fredericksburg, and manufactured by Powers & Weightman.

Of the seven cases, all were cured, and without the occurrence of another chill, after taking the sulphate of cinchona. Prescribed 10 to 15 grains in quotidian, and 15 to 24 in tertian for adults, made into pills with crumb of bread, sometimes sprinkled with pepper, except two. These two were in a poor family, where were eight cases near the same time, and one of them the mother of the other seven, who had no reliable nurse to give her the doses at proper times, and the other a pallid little boy, who had taken but half of his number of doses. As an evidence of his depraved health from the want of quinia or cinchona last fall, I would state, that he was as white as a sheet before having a chill; and, on my second visit, they showed me a hydrocele which had come on in a few days, and which I was informed also made its appearance last fall, and disappeared after the arrest of chills: it is, likewise, now gradually subsiding, though I put him upon carb. fer. precip. and cream tartar. These cases, except one treated in May, were treated since 18th July, in a very poor family, living on the edge of a marshy flat from 100 to 200 yards wide, on Terrible Creek, covered with vegetation waist-high, and inundated by one hard shower of rain knee-deep over its whole area. I saw this family 3d November last year, having been prostrated the whole of the fall (the mother stating that she had been confined to her bed five or six weeks) *starving* for quinine, which promptly arrested their chills. Bad diet and daily exposure to the miasm, and neglect of precautionary remedies, have caused recurrence of one or two chills in this family.

P.S.—The eighth case, a negro woman, has been reported to me by her master. She took eight grains sulphate of cinchona in a tertian, neglecting to take six more during the eighteen hours immediately preceding the period for chill, with the effect of postponing the chill three or four days. The remaining six grains, divided into three doses, were then taken, and she has had no return of chill since—a week or more.

Three of the cases in the family alluded to, have had relapses. One of these, however, was the case treated with sulphate of quinia. On the 27th ult. I sent them a box of pills, composed of 1 grain chinoidine, 1 drop ol. piper. nig. ordering eight or ten to be taken by adults in the intermission, to arrest the chills, and then one or two a-day to prevent relapse.

*Sept. 6th.*—The chinoidine had stopped the chills in all three cases at the first trial, just as promptly as sulphate of quinia or cinchona. The cost of chinoidine is only one-half that of sulphate of cinchona, and one-fourth that of sulphate of quinia. The saving from the use of these cheaper substitutes for one of the *necessaries* of life, in some localities, and in some periods, the source of which is rapidly being exhausted, may be inferred from the fact, that a single mercantile establishment on Staunton River, as I am informed by a physician in its vicinity, has sold within the last twelve months (and I rather think he said during this year) one hundred ounces of sulphate of quinine, generally in half drachm and drachm vials, at fifty cents and a dollar per vial. And I have seen it sold at this rate in former years, mixed often with sulphate of magnesia at that, to make the people think they were getting a large quantity; and this was done (according to the merchant's statement) by the Philadelphia druggist, who "misunderstood his directions, to 'put it up in a showy style,' to mean a large bulk, instead of handsome vials." Whether it had the right weight of quinine, as he said, I never knew, for I refused to allow its use in any of my cases. —*Virginia Medical Journal.*

